# A placebo-controlled study to assess Standardized Field Sobriety Tests performance during alcohol and cannabis intoxication in heavy cannabis users and accuracy of point of collection testing devices for detecting THC in oral fluid

**DOI:** 10.1007/s00213-012-2732-y

**Published:** 2012-05-13

**Authors:** W. M. Bosker, E. L. Theunissen, S. Conen, K. P. C. Kuypers, W. K. Jeffery, H. C. Walls, G. F. Kauert, S. W. Toennes, M. R. Moeller, J. G. Ramaekers

**Affiliations:** 1 Department of Neuropsychology and Psychopharmacology, Faculty of Psychology and Neuroscience, Maastricht University, Maastricht, The Netherlands; 2 Forensic Alcohol and Drug Expert (F.A.D.E.), Burnaby, BC Canada; 3 Department of Pathology, Miller School of Medicine, University of Miami, Miami, FL USA; 4 Institute of Forensic Toxicology University of Frankfurt, Frankfurt/Main, Germany; 5 Saarland University Hospital, Homburg, Germany

**Keywords:** Cannabis, Δ^9^-Tetrahydrocannabinol, THC, SFST, DUID, Oral fluid, Alcohol, Drug, Drug abuse, Cannabinoids

## Abstract

**Rationale:**

Standardized Field Sobriety Tests (SFST) and oral fluid devices are used to screen for driving impairment and roadside drug detection, respectively. SFST have been validated for alcohol, but their sensitivity to impairment induced by other drugs is relatively unknown. The sensitivity and specificity for Δ^9^-tetrahydrocannabinol (THC) of most oral fluid devices have been low.

**Objective:**

This study assessed the effects of smoking cannabis with and without alcohol on SFST performance. Presence of THC in oral fluid was examined with two devices (Dräger Drug Test® 5000 and Securetec Drugwipe® 5).

**Methods:**

Twenty heavy cannabis users (15 males and 5 females; mean age, 24.3 years) participated in a double-blind, placebo-controlled study assessing percentage of impaired individuals on the SFST and the sensitivity of two oral fluid devices. Participants received alcohol doses or alcohol placebo in combination with 400 μg/kg body weight THC. We aimed to reach peak blood alcohol concentration values of 0.5 and 0.7 mg/mL.

**Results:**

Cannabis was significantly related to performance on the one-leg stand (*p* = 0.037). Alcohol in combination with cannabis was significantly related to impairment on horizontal gaze nystagmus (*p* = 0.029). The Dräger Drug Test® 5000 demonstrated a high sensitivity for THC, whereas the sensitivity of the Securetec Drugwipe® 5 was low.

**Conclusions:**

SFST were mildly sensitive to impairment from cannabis in heavy users. Lack of sensitivity might be attributed to tolerance and time of testing. SFST were sensitive to both doses of alcohol. The Dräger Drug Test® 5000 appears to be a promising tool for detecting THC in oral fluid as far as correct THC detection is concerned.

## Introduction

Cannabis is one of the most widely used illicit drugs. About 16.7 million persons or 6.6 % of the USA general population aged 12 and older admitted to past month use (Substance Abuse and Mental Health Services Administration 2010). In Europe, past month users aged 15–64 years was estimated to be 12.5 million (3.7 %) (European Monitoring Centre for Drugs and Drug Addiction 2010). Because of its widespread use, the prevalence of cannabis in the general driving population is also one of the highest after alcohol, with driving under the influence of cannabis (DUIC) being 2.4 % of the general driving population (EMCDDA 2008). Among young drivers, the prevalence of DUIC was even 30 %. This is a serious cause for concern since experimental and epidemiological studies have shown that cannabis impaired driving performance in a concentration-related manner (e.g., Drummer et al. 2004; Grotenhermen et al. 2007; Laumon et al. 2005; Ramaekers et al. 2004; Ramaekers et al. 2009; Ramaekers et al. 2006b).

Oral fluid has gained increasing interest as a valuable matrix for roadside drug testing. In contrast to urine, the advantages include ease of use, noninvasiveness, observable sample collection, difficulty to adulterate, and demonstration of recent drug use. However, at present many immunoassays for detecting Δ^9^-tetrahydrocannabinol (THC) in oral fluid do not have high reliability (Bosker and Huestis 2009; Verstraete 2005). This is mainly due to THC adsorption to the collection device, which makes recovery from the device difficult. In addition, currently used cutoffs of point-of-collection testing devices, although improving, are too high to reliably detect THC in oral fluid. Furthermore, the time window for detection is unknown, and this makes interpretation of the results of oral fluid collection devices difficult. Therefore, controlled drug administration studies are needed (Bosker and Huestis 2009).

Standardized Field Sobriety Tests (SFST) are currently in use in countries such as the USA and Canada for documenting impairment in drivers suspected of driving under the influence. The SFST have been validated for detection of alcohol impairment (Stuster 2006; Stuster and Burns 1998), but their sensitivity to impairment caused by other drugs is relatively unknown. Placebo-controlled studies assessing the effects of single doses of THC, alcohol, and their combination on SFST performance (Papafotiou et al. 2005a; Stough et al. 2006) have reported that performance in the SFST was significantly related to the administration of THC only, and THC with alcohol. The proportion of individuals classified as impaired doubled after THC combined with alcohol. The authors also reported that the use of SFST resulted in the correct classification of up to 73.9 % of participants as either impaired or not. The one-leg stand test of the SFST was found to be the best predictor of impairment. Performance on the SFST however only weakly predicted driving impairment as assessed in a driving simulator (Papafotiou et al. 2005b). SFST also failed to predict actual driving impairment in occasional and heavy cannabis users who received single doses of the synthetic cannabinoid dronabinol. Single doses of dronabinol 10 and 20 mg produced driving impairment comparable to blood alcohol concentration (BAC) levels >0.8 mg/mL as measured in a standardized on-the-road driving test, but went undetected in SFST assessments (Bosker et al. 2012).

The present study was designed to assess the effects of alcohol and THC on SFST performance and to determine the reliability of two point of collection testing devices (Dräger Drug Test® 5000 and Securetec Drugwipe® 5) for detecting THC in oral fluid. It was part of a larger protocol to assess tolerance and cross-tolerance of heavy cannabis users to the impairing effects of THC and alcohol on neurocognitive performance. This data have been published elsewhere (Ramaekers et al. 2011). The latter study showed that neurocognitive performance of heavy, daily cannabis users was impaired during alcohol intoxication, but not after THC smoking. The absence of neurocognitive impairments in heavy cannabis users was interpreted to show behavioral tolerance to the impairing effects of THC. The present part of the study focused on roadside tests for detecting cannabis intoxication in drivers (oral fluid tests and SFST). It was expected that single doses of THC would not affect SFST performance in heavy cannabis users and that THC administration would be detectable in oral fluid.

## Materials and methods

**Fig. 1:**
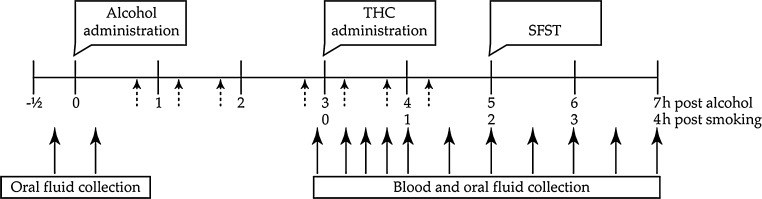
Timeline for drug administration, Standardized Field Sobriety Tests (SFST) performance, and blood and oral fluid collection in hours after alcohol/smoking administration. *Dashed arrows* indicate time points at which booster alcohol doses could be administered on an as-needed basis to achieve steady BAC between 1 and 5 h after onset of drinking

A summary timeline of drug and alcohol administrations, blood and saliva collection, as well as SFST performance is given in Fig. 1.

### Participants

Twenty heavy users with mean (SE) age of 24.3 (1.4) years participated in the study (15 males and 5 females). Participants were recruited by advertisements at Maastricht University and/or at coffee shops and were paid upon completion of the study. Before enrollment, all participants were screened by means of a telephone interview to determine whether they qualified for the study. The inclusion criteria were experience with cannabis (on average smoking on 4 days/week or more during the previous year); free from psychotropic medication; good physical health as determined by a medical examination; absence of any major medical, endocrine, and neurological condition; body mass index between 18 and 28 kg/m^2^; and written informed consent. Furthermore, they needed to test positive for cannabis (THC, 11-OH-THC, and/or THCCOOH) in serum on screening and in urine on every testing day. The exclusion criteria were history of drug abuse or addiction (except cannabis) as assessed by means of a medical questionnaire and drug urine screens, no experience with alcohol, pregnancy or lactation, cardiovascular abnormalities on electrocardiogram, excessive drinking (>25 standard alcoholic consumptions a week), hypertension, history of or current psychiatric disorder, and non-cigarette smokers. If participants met the inclusion criteria, they received a medical history and a drug questionnaire. Finally, participants underwent a medical examination and took part in a training session to get familiar with the tests.

The study was conducted according to the code of ethics on human experimentation established by the Declaration of Helsinki (1964) and amended in Seoul (2008). Approval for the study was obtained from the Medical Ethics committee of the Academic Hospital of Maastricht and Maastricht University. A permit for obtaining, storing, and administering cannabis was obtained from the Dutch drug enforcement administration.

### Study design

The study was conducted according to a double-blind, placebo-controlled three-way design with three alcohol/THC conditions (see Ramaekers et al. 2011). Participants underwent three alcohol-dosing conditions that were designed to achieve steady-state BACs of 0, 0.5, and 0.7 mg/mL during a 5-h time window. The order of alcohol-dosing conditions was counterbalanced across participants. In addition, participants smoked a THC cigarette (400 μg/kg) at 3 h post-onset of alcohol dosing, in each alcohol condition. Alcohol dosing started at 10:30 in the morning with placebo alcohol, 0.5 or 0.7 g/kg alcohol. Additional alcohol booster doses of about 0.1 g/kg or alcohol placebo were given on an as needed basis at approximately every half hour up until 4.5 h after onset of alcohol dosing in order to keep BAC at the desired level. On average, participants received 5.4 additional booster doses containing alcohol (see also Ramaekers et al. 2011). Alcohol was administered as “pure” ethanol (96 %) mixed with orange juice to a volume of 300 mL for the initial dose. Total volumes of booster doses mixed with orange juice were approximately 80 mL. The marijuana cigarettes were prepared beforehand for each individual from stock provided by the Dutch Bureau for Medicinal Cannabis. Marijuana contained 11 % THC, a standard potency for marijuana used recreationally and sold at Dutch pharmacies for medical use. The total amount of cannabis was individually weight calibrated and mixed with tobacco to achieve a standard cigarette size and weight. THC smoking started at 3 h post-onset of alcohol dosing and lasted for 15 min. Participants were instructed to smoke the cigarette according to a standardized procedure (Ramaekers et al. 2006a) in order to minimize the participant’s possibility of dose titration and to increase optimal absorption of THC (inhale for 4 s, hold breath for 10 s, and exhale/break for 15 s). This sequence was repeated until the cigarettes were smoked as completely as possible. The washout period between treatments was at least 4 days.

### Procedure

Participants were asked to refrain from any drugs except cannabis 1 week before the medical examination until study completion. Participants were not allowed to drink alcohol during a 24-h period prior to testing. Participants were allowed to continue their usual cannabis-smoking routine during the study period. Participants were always tested for alcohol and drugs (tetrahydrocannabinol, opiates, amphetamine/ecstasy, benzodiazepines, cocaine, and methamphetamine/ecstasy) in breath and urine, respectively, upon arrival at the laboratory on test days. Treatments were only administered when participants were positive for THC and negative for all other drugs. The procedure on a test day is described elsewhere (Ramaekers et al. 2011). The SFST was performed approximately 2 h after cannabis smoking, which is approximately 5.5 h after the first alcohol dose. In addition, baseline SFST performance was assessed in all individuals on a separate day prior to study treatments.

### Standardized Field Sobriety Tests

The SFST is a battery of three tests administered and evaluated in a standardized manner to obtain validated indicators of impairment after alcohol consumption and establish probable cause for arrest. The tests of the SFST are Horizontal Gaze Nystagmus (HGN), Walk-and-Turn (WAT), and One-Leg Stand (OLS).

HGN is an involuntary jerking of the eye that occurs naturally as the eyes gaze to the side. Under normal circumstances, nystagmus occurs when the eyes are rotated at high peripheral angles. However, when a person is impaired by alcohol, nystagmus is exaggerated and may occur at lesser angles. An alcohol-impaired person will also often have difficulty smoothly tracking a moving object. In the HGN test, the eyes of a participant are observed as the participant follows a slowly moving object, such as a pen, horizontally with his or her eyes. The examiner looks for three indicators of impairment in each eye: if the eye cannot follow a moving object smoothly, distinct jerking at maximum deviation, and angle of onset of jerking within 45° of center. If, between the two eyes, four or more clues appear, the participant likely has a BAC of 0.8 mg/mL or greater and was classified as impaired in the current study. Research has shown that this test allows proper classification of approximately 88 % of participants (Stuster and Burns 1998).

In the WAT test, the participant is directed to take nine steps, heel-to-toe, along a straight line. After taking the steps, the participant must turn on one foot and return in the same manner in the opposite direction. The examiner looks for eight indicators of impairment: participant cannot keep balance while listening to the instructions, participant begins before the instructions are finished, participant stops while walking to regain balance, participant does not touch heel-to-toe, participant steps off the line, participant uses arms to balance, participant makes an improper turn, or takes an incorrect number of steps. Research has indicated that 79 % of individuals who exhibit two or more indicators in the performance of the test will have a BAC of 0.8 mg/mL or greater (Stuster and Burns 1998). When participants showed two or more signs of impairment, he/she was classified as being impaired in the current study.

In the OLS test, the participant is instructed to stand with one foot approximately 6 in. (15 cm) off the ground and count aloud from 1,000 (1,000, 1,001, 1,002, etc.) for 30 s. The examiner looks for four indicators of impairment: swaying while balancing, using arms to balance, hopping to maintain balance, and putting the foot down. Research has indicated that 83 % of individuals who exhibit two or more indicators in the performance of the test will have a BAC of 0.8 mg/mL or greater (Stuster and Burns 1998). The time it took to count to 1,030 is also noted. Participants showing two or more indicators of impairment were classified as being impaired on this test.

In addition, an overall SFST score was generated. Participants were classified as impaired overall whenever he/she showed impairments on two out of three SFST. Percentages of participants showing impairment on HGN, WAT, OLS, and overall SFST performance were the dependent variables.

### Pharmacokinetic assessments

Oral fluid and blood samples (6 mL) were collected at baseline, 15, 30, 45, and 60 min during the first hour after smoking and subsequently every 30 min between 1 and 4 h after smoking. Oral fluid from half of the participants was collected using the Dräger Drug Test® 5000 and the other half with the Securetec Drugwipe® 5 device. With the Securetec Drugwipe® 5 two results are produced: one for oral fluid taken from the cheek and the other from the tongue. The cutoff for cannabinoids as stated by the manufacturers of the Dräger Drug Test® 5000 is 5 ng/mL and of the Securetec Drugwipe® 5 30 ng/mL. The blood sample was centrifuged, and the resulting serum was frozen at −20°C until analysis. THC, 11-OH-THC, and THCCOOH concentrations were determined afterwards (Toennes et al. 2008). BAC was assessed in serum at regular intervals (see Toennes et al. 2011) prior to SFST performance.

### Statistical analysis

All statistical analyses were performed using SPSS 18.0 for Mac. For each SFST and for the overall impairment score, separate chi-square (*χ* ^2^) tests were performed to determine whether a relationship existed between SFST performance and treatment conditions. SFST performance in each alcohol condition (0.5 mg/mL + THC and 0.7 mg/mL + THC) and in the THC-only condition (alcohol placebo) was compared to baseline. In case of a significant treatment effect, Spearman’s coefficient (*ρ*) was calculated to determine the strength and direction of the relationship between treatment condition and SFST performance. A positive relationship indicates that impairment increases with increasing dose and a negative relationship that impairment decreases with increasing dosage.

For the oral fluid tests, the percentages of false negatives for THC (i.e., oral fluid tests that indicated the participant was negative for THC divided through the total number of oral fluid tests at every time point with all treatment conditions collapsed) were calculated. The sensitivity was calculated by dividing true positives (i.e., oral fluid tests that indicated a participant was positive for THC), through true positives + false negatives.

## Results

### Dropouts

Two participants dropped out after the first treatment condition for reasons unrelated to the study, and one participant had missing data at baseline. Available data entered statistical analysis.

### Standardized Field Sobriety Tests

Percentages of impaired participants on the SFST are displayed in Table 1. Cannabis alone was significantly related to OLS impairment (*χ* ^2^ = 4.364, *df* = 1, *p* = 0.037), and a trend was shown for HGN (*χ* ^2^ = 3.399, *df* = 1, *p* = 0.065). Both relationships were positive (*ρ* = 0.3, *p* = 0.019; *ρ* = 0.3, *p* = 0.034, respectively).

**Table 1:** Individual impairment score on HGN, WAT, OLS and overall SFST performance during baseline and under the influence of THC in combination with alcohol placebo, 0.5 mg/mL BAC and 0.7 mg/mL BAC

Participant	Baseline (*N* = 19)				THC + alcohol placebo (*N* = 20)				THC + 0.5 mg/mL BAC (*N* = 19)				THC + 0.7 mg/mL BAC (*N* = 18)			
	HGN	WAT	OLS	total	HGN	WAT	OLS	total	HGN	WAT	OLS	total	HGN	WAT	OLS	total
1	–	–	–	–	0	0	0	0	1	0	1	1	0	1	1	1
2	0	1	1	1	0	1	1	1	0	0	0	0	0	0	0	0
3	0	1	1	1	0	0	1	0	0	1	1	1	0	1	1	1
4	0	1	0	0	0	0	0	0	0	1	0	0	0	1	0	0
5	0	0	0	0	0	0	0	0	0	0	0	0	0	1	0	0
6	0	1	0	0	0	1	1	1	0	1	0	0	0	1	0	0
7	0	0	0	0	0	1	0	0	0	1	0	0	1	1	0	1
8	0	0	0	0	1	0	0	0	0	0	1	0	1	1	1	1
9	0	1	0	0	0	0	0	0	0	0	0	0	0	0	0	0
10	0	1	0	0	0	0	1	0	0	0	1	0	0	1	0	0
11	0	1	0	0	0	0	1	0	0	1	0	0	0	0	0	0
12	0	0	0	0	0	0	0	0	0	0	1	0	0	0	0	0
13	0	1	1	1	1	1	1	1	1	1	1	1	1	1	1	1
15	0	0	0	0	0	0	0	0	0	0	0	0	–	–	–	–
16	0	0	0	0	0	1	1	1	0	0	1	0	1	1	1	1
17	0	1	0	0	0	0	0	0	0	0	0	0	1	0	0	0
19	0	1	0	0	0	1	1	1	0	1	1	1	0	1	1	1
20	0	0	1	0	0	0	1	0	0	0	1	0	0	1	1	1
21	0	0	0	0	0	1	1	1	0	0	1	0	0	0	1	0
23	0	1	0	0	1	0	0	0	–	–	–	–	–	–	–	–
Total (%)	0	58	21	16	15	35	50	30	11	37	53	21	28	67	44	44

*HGN* Horizontal Gaze Nystagmus, *WAT* Walk-and-Turn, *OLS* One-Leg Stand, *SFST* Standardized Field Sobriety Tests, *BAC* blood alcohol concentration

The results showed that the combinations of alcohol and cannabis were significantly related to HGN impairment (*χ* ^2^ = 7.110, *df* = 2, *p* = 0.029). A trend was shown on overall SFST performance (*χ* ^2^ = 4.939, *df* = 2, *p* = 0.085) and OLS impairment (*χ* ^2^ = 5.249, *df* = 2, *p* = 0.072). All correlations were positive (*ρ* = 0.3, *p* = 0.004; *ρ* = 0.3, *p* = 0.018; *ρ* = 0.2, *p* = 0.043, respectively).

### Pharmacokinetic measures

The percentages of false negatives and the sensitivity of the Dräger Drug Test® 5000 and Securetec Drugwipe® 5 and the associated concentrations of THC, 11-OH-THC, and THCCOOH in serum are shown in Table 2. The Dräger Drug Test® 5000 generally performed well after acute administrations of THC. The percentages of false negatives and sensitivities ranged from 0–10 % and 90–100 %, respectively, between 15 min after smoking and 3 h after smoking. However, the test was less accurate at the baseline measures before drinking/smoking. At baseline, the percentages of false negatives ranged from 22–48 % and sensitivities from 52–78 %, while mean THC serum concentration was 7.1 ng/mL (range, 0–20 ng/mL). The performance of the Securetec Drugwipe® 5 was less accurate. Performance was best 15 min after smoking (8 % false negatives, sensitivity 92 %). As time after smoking progressed, the percentages of false negatives increased and sensitivities were low. At baseline, accuracy was low as well.

**Table 2:** Percentages of false negatives (FN) and the sensitivity (SN) of the Dräger Drug Test® 5000 and Securetec Drugwipe® 5 (from cheek and tongue) and mean THC, 11-OH-THC, and THCCOOH concentrations (nanograms per milliliter) in serum relative to time after smoking collapsed over treatment conditions

Time after smoking (h)	Dräger Drug Test® 5000		Securetec Drugwipe® (cheek)		Securetec Drugwipe® (tongue)		THC	THCCOOH	11-OH-THC
	FN	SN	FN	SN	FN	SN			
Baseline	–	–	–	–	–	–	7.1 (1.4)	50.1 (8.4)	2.9 (0.7)
−03:15	28	72	48	52	50	50	–	–	–
−02:45	22	78	46	54	54	46	–	–	–
−00:05	48	52	58	42	54	46	9.1 (1.7)	69.7 (8.5)	5.1 (1.0)
00:15	3	97	8	92	8	92	101.1 (6.1)	98.3 (10.4)	17.5 (1.5)
00:30	3	97	29	71	13	88	47.4 (3.4)	101.8 (11.0)	15.2 (1.3)
00:45	6	94	46	54	38	63	30.1 (2.0)	95.0 (10.1)	12.5 (1.0)
01:00	3	97	46	54	35	65	22.1 (1.4)	90.4 (10.3)	10.8 (0.9)
01:30	0	100	42	58	33	67	18.6 (1.2)	85.2 (10.0)	9.3 (0.8)
02:00	3	97	42	58	38	63	13.1 (1.0)	80.5 (9.6)	7.5 (0.7)
02:30	3	97	54	46	50	50	10.3 (0.8)	76.4 (9.7)	6.2 (0.6)
03:00	10	90	52	48	38	63	8.2 (0.7)	68.3 (8.9)	5.1 (0.6)
03:30	18	82	50	50	43	57	8.5 (0.7)	66.8 (7.7)	5.1 (0.6)
04:00	7	93	52	48	46	54	8.0 (0.9)	66.6 (8.3)	4.7 (0.5)

Mean BAC concentrations during SFST performance after THC, THC + alcohol 0.5 mg/mL, and THC + alcohol 0.7 mg/mL were 0, 0.37, and 0.51 mg/mL, respectively. Mean THC concentrations during these conditions were 13.4, 14.4, and 11.6 ng/mL, respectively.

## Discussion

This study was designed to assess the effects of THC and the combination of alcohol and THC on SFST performance. Two oral fluid tests (Dräger Drug Test® 5000 and Securetec Drugwipe® 5) were assessed for their accuracy in determining the presence of THC.

The SFST was mildly sensitive to the effects of cannabis alone. A dose of 400 μg/kg body weight THC significantly increased the percentage of participants displaying impairments in OLS compared to baseline performance from 21 to 50 %. THC also increased percentage of individuals showing impairment on HGN from 0 to 15 %, relative to baseline, but this change only approached statistical significance. WAT and the overall score on SFST did not discriminate between THC and baseline. These findings appear in line with previous studies that have reported a relation between impairment on the SFST and presence of THC in blood. A study that assessed which signs of the Drug Evaluation and Classification evaluations predicted various drug categories (including cannabis) at best showed that OLS contributed significantly to the prediction, but HGN and WAT did not (Porath-Waller et al. 2009). Papafotiou et al. (2005a) assessed SFST performance in 40 healthy participants who received low and high doses of THC in a placebo-controlled study. On average, blood THC concentrations obtained after the highest dose were comparable to serum THC concentrations achieved in the present study after smoking cannabis. Yet, THC significantly affected performance on OLS, HGN, and WAT and appeared to be more prominent as compared to the current study. For example, in that study THC produced impairments on overall SFST performance in up to 50 % of the participants (Papafotiou et al. 2005a) but in only 30 % of the participants of the present study. These differences may be explained in terms of differences in cannabis use history. In the study by Papafotiou et al. (2005a), the reported frequency of cannabis use of the participants varied from once a week to once every 2–6 months. The present study however only included heavy cannabis users, who smoked cannabis on at least four occasions per week. Previous studies demonstrated that heavy cannabis users develop tolerance to the impairing effects of THC on neurocognitive measures (Hart et al. 2001; Ramaekers et al. 2011). It is likely that many of the participants who participated in the present study, in part or in total, developed tolerance to the impairing effects of THC as well. In such a scenario, the failure of the SFST to demonstrate robust effects of THC is not necessarily an indicator of poor sensitivity, but may reflect the chronic cannabis use of the participants.

Alternatively, one might argue that SFST were conducted too late after cannabis administration. SFST were not performed directly after smoking when impairments or THC concentrations can be expected to be maximal. Instead, SFST were performed 2 h after smoking in the present study, when THC impairments are on the decline, as shown in occasional users. It should be noted however that performance impairment has repeatedly been shown to last for 3–4 h after smoking THC (e.g., Ramaekers et al. 2009, 2006a). In the present study, ratings of subjective high were also significantly elevated at 2 h after smoking as reported elsewhere (Ramaekers et al. 2011). Likewise, average THC concentrations during SFST performance were above THC threshold levels above which performance impairments are expected to appear in occasional users (Ramaekers et al. 2006b). In other words, the SFST were conducted well within the established “impairment window” of 3–4 h post-smoking, even though the level of impairment was submaximal. The relative lack of sensitivity of SFST for cannabis effects in the present study thus cannot be explained by a total lack of cannabis intoxication at the time of testing. Still, it cannot be excluded that SFST might have been more sensitive to the effects of THC if conducted right after smoking.

In general, the present data indicate that SFST were mildly sensitive to the effects of THC depending on dose and cannabis use history. It is noteworthy, however, that SFST were unable to discriminate performance impairments produced by dronabinol, a synthetic cannabinoid (Bosker et al. 2012). In this placebo-controlled study, occasional and heavy users received oral doses of dronabinol that produced significant on-the-road driving impairments that were comparable to those observed after BACs >0.8 mg/mL. THC concentrations after oral dronabinol however were much lower (<10 ng/mL) than those achieved after smoking cannabis. Also, THC concentrations during SFST testing were half that in the dronabinol study compared to the present study. This fits with the general conclusion that SFST are mildly sensitive to the impairing effects of THC, but that impairments may go undetected in some individuals, particularly at lower THC concentrations.

THC with alcohol generally increased the number of individuals displaying impairment on HGN, OLS, and total SFST score. Relative to baseline, percentages of impaired individuals increased after both alcohol combinations with THC in a dose-dependent manner. HGN was the only measure that did not reveal any impairment in participants during baseline. It should be remembered here that all participants included were heavy cannabis users who were always positive for THC, also during baseline. On average, baseline THC levels were 7.1 ng/mL. HGN may thus be an interesting parameter to separate residual THC use from recent THC intoxication when OLS and overall SFST performance show impairment. In general, impairments observed after the combination of THC and alcohol are most likely attributable to alcohol since most of the current participants may have developed tolerance to the impairing effects of THC on performance. As such, the present data confirm the sensitivity of the SFST for alcohol-induced impairment.

The point of collection testing devices provided mixed results. The Dräger Drug Test® 5000 generally performed quite well and only produced false negative in 6 % of the measurements during 2.5 h after smoking. Securetec Drugwipe® 5 however performed poorly in detecting THC. The rate of false negatives was already 8 % 15 min after smoking and rapidly increased to about 40–50 % within the hour. The difference in sensitivity between both devices may well be related to differences in cutoff levels which are 5 ng/mL in the Dräger Drug Test® 5000 and 30 ng/mL in the Securetec Drugwipe® 5 according to the manufacturers. It should be noted that in this study we did not determine the rate of false positives for both devices because our participants were never drug free. Even during baseline, low levels of THC were present in all participants. A recent roadside study reported about 2 % false positives for the Dräger Drug Test® 5000 and about 3 % false negatives. The sensitivity, specificity, and accuracy were 93, 71, and 90 %, respectively (Wille et al. 2010). Together, these data indicate that point of collection testing devices for detecting THC in oral fluid are making large improvements relative to some of the previous devices that were on the market (Bosker and Huestis 2009; Ramaekers et al. 2006b). Still it has been argued in the ROSITA-2 project that the sensitivity and specificity of oral fluid devices should be higher than 90 % (Verstraete and Raes 2006). According to these criteria, the Dräger Drug Test® 5000 would still fall short on specificity (Wille et al. 2009). However, compared to the alternative matrix urine, oral fluid drug concentrations have a better correlation with blood concentrations and recent use of cannabis. It is therefore still the preferable matrix for evaluating THC presence in drivers (Bosker and Huestis 2009; Toennes et al. 2005).

Taken together, the results indicated that the SFST were mildly sensitive to THC use in heavy users, probably because many of the participants have developed behavioral tolerance to THC-induced impairments. SFST were sensitive to low levels of alcohol in combination with THC as indicated by increments in the number of participants rated as impaired on HGN, OLS, and total SFST score. The Dräger Drug Test® 5000 achieved a high sensitivity for THC after acute THC administration in the present study, but the sensitivity in the case of the Securetec Drugwipe® 5 was low.

## References

[CR1] Bosker WM, Huestis MA (2009). Oral fluid testing for drugs of abuse. Clin Chem.

[CR2] Bosker WM, Kuypers KPC, Theunissen EL, Surinx A, Blankespoor RJ, Skopp G, Jeffery WK, Walls HC, Ramaekers JG (2012) Medicinal THC (dronabinol) impairs on-the-road driving performance of occasional and heavy cannabis users but is not detected in Standardized Field Sobriety Tests. Addiction. doi:10.1111/j.1360-0443.2012.03928.x 10.1111/j.1360-0443.2012.03928.x 22553980

[CR3] Drummer OH, Gerostamoulos J, Batziris H, Chu M, Caplehorn J, Robertson MD, Swann P (2004). The involvement of drugs in drivers of motor vehicles killed in Australian road traffic crashes. Accid Anal Prev.

[CR4] EMCDDA (2008). EMCDDA Insights Series No 8: drug use, impaired driving and traffic accidents.

[CR5] European Monitoring Centre for Drugs and Drug Addiction (2010). Annual report 2010: the state of the drugs problem in Europe.

[CR6] Grotenhermen F, Leson G, Berghaus G, Drummer OH, Kruger HP, Longo M, Moskowitz H, Perrine B, Ramaekers JG, Smiley A, Tunbridge R (2007). Developing limits for driving under cannabis. Addiction.

[CR7] Hart CL, van Gorp W, Haney M, Foltin RW, Fischman MW (2001). Effects of acute smoked marijuana on complex cognitive performance. Neuropsychopharmacology.

[CR8] Laumon B, Gadegbeku B, Martin JL, Biecheler MB (2005). Cannabis intoxication and fatal road crashes in France: population based case–control study. BMJ.

[CR9] Substance Abuse and Mental Health Services Administration (2010). Results from the 2009 National Survey on Drug Use and Health: volume I. Summary of national findings.

[CR10] Papafotiou K, Carter JD, Stough C (2005). An evaluation of the sensitivity of the Standardised Field Sobriety Tests (SFSTs) to detect impairment due to marijuana intoxication. Psychopharmacology (Berl).

[CR11] Papafotiou K, Carter JD, Stough C (2005). The relationship between performance on the standardised field sobriety tests, driving performance and the level of Delta9-tetrahydrocannabinol (THC) in blood. Forensic Sci Int.

[CR12] Porath-Waller AJ, Beirness DJ, Beasley EE (2009). Toward a more parsimonious approach to drug recognition expert evaluations. Traffic Inj Prev.

[CR13] Ramaekers JG, Berghaus G, van Laar M, Drummer OH (2004). Dose related risk of motor vehicle crashes after cannabis use. Drug Alcohol Depend.

[CR14] Ramaekers JG, Kauert G, van Ruitenbeek P, Theunissen EL, Schneider E, Moeller MR (2006). High-potency marijuana impairs executive function and inhibitory motor control. Neuropsychopharmacology.

[CR15] Ramaekers JG, Moeller MR, van Ruitenbeek P, Theunissen EL, Schneider E, Kauert G (2006). Cognition and motor control as a function of Delta9-THC concentration in serum and oral fluid: limits of impairment. Drug Alcohol Depend.

[CR16] Ramaekers JG, Kauert G, Theunissen EL, Toennes SW, Moeller MR (2009). Neurocognitive performance during acute THC intoxication in heavy and occasional cannabis users. J Psychopharmacol.

[CR17] Ramaekers JG, Theunissen EL, de Brouwer M, Toennes SW, Moeller MR, Kauert G (2011). Tolerance and cross-tolerance to neurocognitive effects of THC and alcohol in heavy cannabis users. Psychopharmacology (Berl).

[CR18] Stough C, Boorman M, Ogden E, Papafotiou K (2006). An evaluation of the Standardised Field Sobriety Tests for the detection of impairment associated with cannabis with and without alcohol.

[CR19] Stuster J (2006). Validation of the standardized field sobriety test battery at 0.08 % blood alcohol concentration. Hum Factors.

[CR20] Stuster J, Burns M (1998) Validation of the Standardized Field Sobriety Test battery at BACs below 0.10. US Department of Transportation, National Highway Traffic Safety Administration, DOT-HS-808-839, Washington, DC

[CR21] Toennes SW, Kauert GF, Steinmeyer S, Moeller MR (2005). Driving under the influence of drugs—evaluation of analytical data of drugs in oral fluid, serum and urine, and correlation with impairment symptoms. Forensic Sci Int.

[CR22] Toennes SW, Ramaekers JG, Theunissen EL, Moeller MR, Kauert GF (2008). Comparison of cannabinoid pharmacokinetic properties in occasional and heavy users smoking a marijuana or placebo joint. J Anal Toxicol.

[CR23] Toennes SW, Schneider K, Kauert GF, Wunder C, Moeller MR, Theunissen EL, Ramaekers JG (2011). Influence of ethanol on cannabinoid pharmacokinetic parameters in chronic users. Anal Bioanal Chem.

[CR24] Verstraete A (2005). Oral fluid testing for driving under the influence of drugs: history, recent progress and remaining challenges. Forensic Sci Int.

[CR25] Verstraete A, Raes E (2006). Rosita-2 project: final report.

[CR26] Wille SM, Raes E, Lillsunde P, Gunnar T, Laloup M, Samyn N, Christophersen AS, Moeller MR, Hammer KP, Verstraete AG (2009). Relationship between oral fluid and blood concentrations of drugs of abuse in drivers suspected of driving under the influence of drugs. Ther Drug Monit.

[CR27] Wille SM, Samyn N, Ramirez-Fernandez Mdel M, De Boeck G (2010). Evaluation of on-site oral fluid screening using Drugwipe-5(+), RapidSTAT and Drug Test 5000 for the detection of drugs of abuse in drivers. Forensic Sci Int.

